# The association between e-cigarette use among Chinese college students and lung function and physical activity: A cross-sectional study

**DOI:** 10.18332/tid/213718

**Published:** 2025-12-20

**Authors:** Diwen Wang

**Affiliations:** 1 Faculty of Biological Sciences, University of Leeds, Leeds, United Kingdom

**Keywords:** electronic cigarettes, respiratory symptoms, impulse oscillometry, university students

## Abstract

**INTRODUCTION:**

Electronic cigarettes have gained widespread popularity among young adults, yet their respiratory health impacts remain incompletely understood. This cross-sectional investigation aimed to examine associations between e-cigarette use and respiratory symptoms, pulmonary function, and physical activity among Chinese university students.

**METHODS:**

This cross-sectional study was conducted at a university in China from January to August 2024. The study recruited 122 participants aged 18–30 years from a university setting, comprising 60 regular e-cigarette users with ≥1-month consistent usage and 62 tobacco-naive controls. Comprehensive assessments included respiratory symptom evaluation using the Dyspnoea-12 questionnaire, pulmonary function testing via spirometry and impulse oscillometry, fractional exhaled nitric oxide measurement, and physical activity assessment through standardized questionnaires and objective monitoring. Statistical analyses employed independent samples t-tests, Mann-Whitney U tests, and multivariable linear regression models with significance set at p<0.05.

**RESULTS:**

The investigation revealed significantly elevated respiratory symptom burden among e-cigarette users compared to controls, with Dyspnoea-12 scores reaching 2.0 versus 0 points (mean difference=2.0; 95% CI: 0.5–3.5, p=0.008, Cohen's d=0.52). Analysis suggested a potential dose-response pattern with symptom escalation from 1.0 points in short-term users (1–3 months) to 3.5 points in long-term users (7–12 months, p=0.018). Impulse oscillometry detected subtle but significant increases in peripheral airway resistance (0.31 ± 0.07 vs 0.29 ± 0.06 kPa/L.s, p=0.045) and frequency-dependent resistance changes (p=0.028), among e-cigarette users compared to controls, indicating small airway dysfunction despite preserved conventional spirometric parameters. Physical activity levels remained comparable between groups across multiple assessment domains. Multivariable regression confirmed that e-cigarette use was independently associated with elevated respiratory symptoms (β=3.12; 95% CI: 1.01–5.23, p=0.004).

**CONCLUSIONS:**

These findings demonstrate that e-cigarette use among university students is associated with clinically meaningful respiratory symptom burden and early airway dysfunction, supporting the need for further longitudinal studies and the development of symptom-based surveillance systems in academic settings.

## INTRODUCTION

Electronic cigarettes (e-cigarettes) have emerged as rapidly proliferating tobacco products worldwide, particularly among young adults aged 18–30 years. Recent systematic reviews demonstrate that global e-cigarette prevalence among youth has reached substantial levels, with comprehensive meta-analyses indicating usage rates of 15–25% among college students^1^. Medical university students, representing a highly educated demographic, show particularly concerning usage patterns, with studies revealing that approximately 20.9% have experimented with e-cigarettes at least once^2^. Despite widespread perceptions of reduced harm compared to traditional cigarettes, college students’ understanding of e-cigarette risks remains inadequate, with many underestimating the addictive potential and long-term health consequences^3^. E-cigarette fluids possess serious health issues since detailed reviews identify complicated mixtures of nicotine salts, propylene glycol, glycerin, and flavoring compounds^4^. Even though e-cigarettes circumvent combustion product exhalants seen in combustible cigarettes, their aerosol contains many toxic compounds including formaldehyde, acetaldehyde, and heavy elements like lead, nickel, and chromium^5^. Experimental studies establish that repeated daily exposure of carbonyl compounds varies substantially with concentration of nicotine and device features and can elevate the potency of cancer risk^6^.

Existing literature lacks the detailed respiratory health outcomes for e-cigarette consumption among general youth populations^7,8^ underlying mechanisms for such. Systematic reviews assessing respiratory outcomes among non-smoker populations outline sparse evidence for serious outcomes, but infer prospective damage for mild presentations of respiratory symptoms such as wheezing and coughing^7^. Underlying mechanisms for such outcomes occur through multi-faceted interaction among flavoring agents and respiratory epithelium, and isolated compounds such as cinnamaldehyde and diacetyl are recognized for cytotoxic and inflammatory actions^8^. Standard spirometry, although consistently employed for the assessment of pulmonary function, reveals sparse sensitivity for the identification of dysfunctional airways following short-term exposure. Impulse oscillometry (IOS) possesses higher sensitivity for the identification of small airway dysfunction of normal traditional pulmonary function parameters among symptomatic populations^9^. Investigations into early signs of chronic obstructive pulmonary disease indicate the prospective utility of IOS for the identification of subclinical respiratory impairment^10^, for which established methodological standards underpin its clinical utility^11^. The association between e-cigarette consumption and exertion yields paradoxical results, as evidence indicates that e-cigarette consumption among youth populations parallels the preservation or even augmentation of exertion thresholds, an anomalous contrast with the established negative correlations for traditional tobacco consumption^12^.

The present cross-sectional study addresses knowledge gaps by systematically examining associations between e-cigarette use and pulmonary function, physical activity, and respiratory symptoms among Chinese university students aged 18–30 years. While previous surveillance data demonstrate increasing e-cigarette prevalence among American adolescents and young adults^13^, limited research has specifically investigated these relationships using sensitive pulmonary function assessment methods in Chinese populations. Recent longitudinal studies utilizing the Population Assessment of Tobacco and Health data, reveal significant associations between youth e-cigarette use and functionally important respiratory symptoms^14^, yet comprehensive evaluations incorporating impulse oscillometry and physical activity assessments remain scarce. This investigation employs impulse oscillometry and comprehensive physical activity assessments to identify early indicators of health changes associated with e-cigarette use. The study aims to examine associations between e-cigarette use and pulmonary function, physical activity, and respiratory symptoms among Chinese university students aged 18–30 years, providing essential scientific evidence for public health education in academic settings.

## METHODS

### Study design, setup and participants

**Figure 1:**
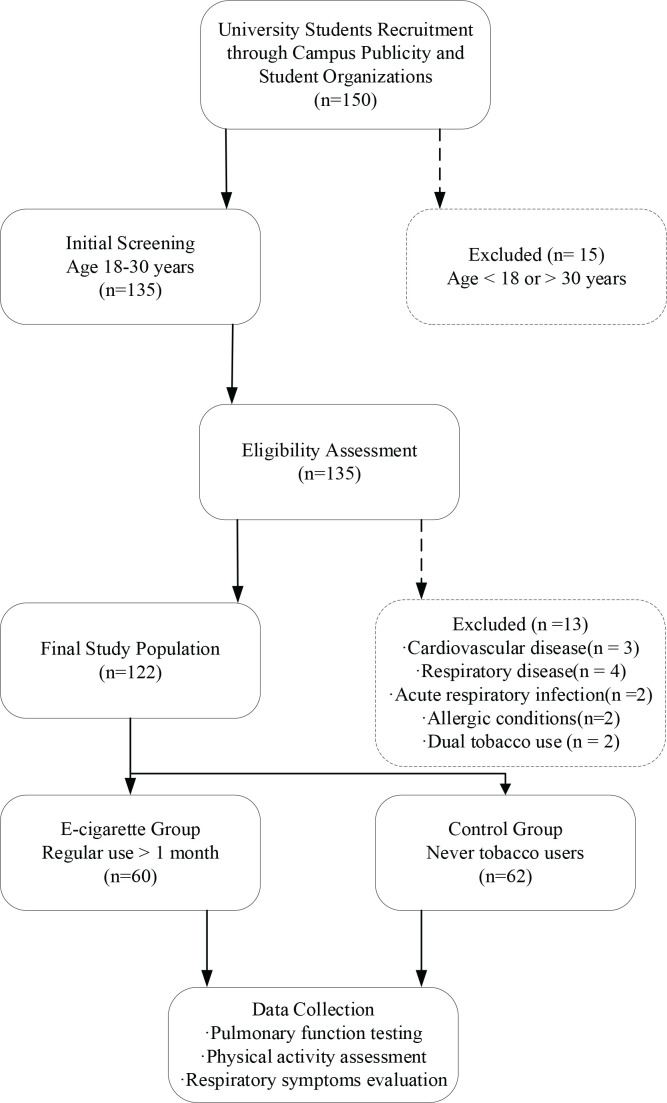
Study flow diagram illustrating participant recruitment, screening, and group allocation process in a cross-sectional study among Chinese university students, China, January–August 2024

This cross-sectional observational study was conducted in accordance with the Strengthening the Reporting of Observational Studies in Epidemiology (STROBE) statement^15^ and implemented at a university in [specific city/province], China, from January to August 2024. Sample size calculations were based on preliminary Dyspnoea-12 score differences with an anticipated effect size of d=0.5, derived from preliminary data and previous literature on respiratory symptoms in young adult populations^16,17^, alpha level of 0.05, and power of 1-β=0.90, accounting for a 10% attrition rate, yielding a requirement of approximately 60 participants per group. The final enrollment comprised 60 participants in the e-cigarette group and 62 participants in the control group, for a total sample of 122 individuals. University students aged 18–30 years were recruited through campus publicity and student organization networks. The e-cigarette group comprised regular users with ≥1 month of consistent usage, while the control group consisted of individuals who had never used any tobacco products. Exclusion criteria encompassed participants with cardiovascular or respiratory disease history, acute respiratory infections within the preceding four weeks, diagnosed allergic conditions, traditional tobacco use history, or dual product usage patterns. The recruitment and screening process, as illustrated in Figure 1, resulted in the enrollment of 60 participants in the e-cigarette group and 62 participants in the control group, achieving the target sample size for adequate statistical power.

### Variables and data sources

This investigation employed a comprehensive data collection framework encompassing multiple outcome measures and exposure variables. The main outcome variables were respiratory symptoms evaluated by the Dyspnoea-12 questionnaire (a validated 12-item breathlessness assessment tool with scores ranging from 0 to 36, where higher scores indicate greater dyspnea severity), pulmonary function parameters measured by spirometry (forced expiratory volume in one second [FEV_1_], forced vital capacity [FVC]) and impulse oscillometry, and physical activity evaluated by both the International Physical Activity Questionnaire-Short Form (IPAQ-SF)^18^ and objective step count assessed by accelerometry. The variable of exposure was detailed e-cigarette usage that was recorded through standardized questionnaires, including duration, frequency per day, preferred device type, and nicotine concentration level (self-reported based on product packaging information). Thorough characterization of e-cigarette use involved the evaluation of pod-based versus tank-based models, preferred flavors, and puffing habits to establish proper exposure classification. Potential confounding variables were methodically recorded for controlling for demographic and behavioral factors that could confound the association of e-cigarette use with health outcomes. These participant covariates included age, sex, body mass index calculated from measured height and weighed height for age and sex adjustment, education level, sleep quality evaluated through questionnaire using the Pittsburgh Sleep Quality Index^19^, eating habits assessed via a valid food frequency questionnaire, and environmental exposure history such as secondhand smoke exposure and occupational respiratory hazard.

## METHODS

This research utilized standardized measurement protocols across various fields of physiology. Pulmonary function testing utilized the Jaeger Vyntus system for impulse oscillometry and spirometry, and fractional exhaled nitric oxide (FeNO) assessment was carried out by using the NIOX VERO device, and all such procedures were strictly carried out as per American Thoracic Society and European Respiratory Society guidelines^11^. Physical activity assessment integrated subjective and objective approaches, namely the International Physical Activity Questionnaire-Short Form (IPAQ-SF) to measure seven-day physical activity patterns and mobile phone-based pedometer measures of average daily steps during the study period. Functional capacity tests also included standardized grip strength measurements with a digital dynamometer with the best of three measures, and cardiovascular endurance testing by a modified six-minute walk test of 25 meters. All measurements were conducted by trained research staff employing standard protocols in order to achieve consistency and reliability across participants, with quality control checks incorporated into the data-gathering process in order to limit measurement error and enhance the precision of outcome measures.

### Bias control

This study employed extensive bias control measures to maximize the validity and reliability of study results. Selection bias was reduced through the use of consecutive sampling methodology and strict compliance with predetermined criteria for participant inclusion and exclusion, allowing for representative participant recruitment without systematic selection biases. Information bias was managed through the use of standardized training procedures for all personnel involved in conducting measurements, with ongoing calibration and maintenance of testing instrumentation for the purpose of ensuring measurement precision and consistency over the period of the study. Pre-testing conditions were held constant for the purpose of reducing potential confounding impacts upon outcome measurements, with participants required to abstain from strenuous physical exertion for the period of 24 hours leading up to testing sessions and refrain from use of e-cigarettes upon the day of assessment. These systematic bias control procedures were employed for the purpose of bolstering the internal validity of associations observed and the minimization of spurious results based upon methodological limitations, thus maximizing the overall scientific rigor and interpretability of results within the framework of e-cigarette health impact studies.

### Ethical considerations

Ethical approval for this study was granted by the Human Ethics Review Committee and conformed strictly with the principles of the Declaration of Helsinki for human research ethics. Written informed consent was obtained from all participants after detailed explanation of the study objectives, procedures, potential harms, and anticipated benefits. Voluntary participation was stressed, and participants were entitled to drop out of the study at any point without losing their entitlement to routine health and academic services. Measures for protecting the data involved anonymous coding strategies for the protection of the participant, with the scientific data used solely for the purpose of science and stored securely according to institutional guidelines for data security. The study design emphasized the importance of participant safety and dignity and responded meaningfully to significant public health questions for e-cigarette health risk among young adults. This ethical framework ensured that the study contributed meaningfully to evidence-informed policy development and held the highest standards of participant protection and scientific integrity throughout the research process.

### Statistical analysis

Descriptive statistics are presented as mean ± standard deviation (SD) for normally distributed continuous variables, median and interquartile range (IQR) for non-normally distributed continuous variables, and frequencies and percentages for categorical variables. The Dyspnoea-12 questionnaire has demonstrated high internal consistency (Cronbach’s α>0.90) and test-retest reliability in previous validation studies^16,17^. The IPAQ-SF has shown acceptable reliability (intraclass correlation coefficient=0.80) for assessing physical activity^18^. The NIOX VERO device has demonstrated high precision with coefficients of variation <5% for repeated measurements. All enrolled participants completed the full battery of assessments with no missing data; therefore, no imputation procedures were required.

Statistical analysis was performed using SPSS version 29.0, with the level of significance set at α=0.05. The normality of continuous variables was assessed using the Shapiro-Wilk test and visual inspection of Q-Q plots. Variables that deviated from normal distribution (Dyspnoea-12 scores, FeNO values, IPAQ-SF scores, usage duration) were analyzed using non-parametric tests or presented as median (IQR). Independent samples t-tests or Mann-Whitney U tests were used for analysis of continuous variables with respect to their data distributions, and categorical variables were analyzed by the use of the chi-squared tests. The effect size was estimated by the use of Cohen’s d for the practical significance of the differences observed. Confounding variables were selected based on biological plausibility and previous literature demonstrating associations with both e-cigarette use and respiratory outcomes, including age, gender, body mass index, sleep quality, and environmental exposures. Stratification analyses were carried out by gender and age group. Interaction terms between e-cigarette use and gender (e-cigarette × gender) and between e-cigarette use and age group (e-cigarette × age) were tested in multivariable models with the same significance threshold (α=0.05). Multivariable linear regression models were developed to control for confounding variables and establish dose-effect associations between e-cigarette exposure parameters and health outcomes. No transformations were applied to dependent or independent variables in regression models; instead, robust standard errors were used when assumption violations were detected. Assumptions for the models were tested using residual analysis.

The primary outcome was respiratory symptom severity assessed by Dyspnoea-12 total score. Secondary outcomes included impulse oscillometry parameters (R5; R5-R20), spirometric measures (FEV_1_, FVC, FEV_1_/FVC, FEF25-75), FeNO levels, and physical activity measures. Given the exploratory nature of this study with pre-specified hypotheses for each outcome, no corrections for multiple comparisons were applied, and results should be interpreted with appropriate caution. Sensitivity analyses were conducted to verify the robustness of primary findings, including robustness testing through exclusion of potential outliers (defined as Dyspnoea-12 scores exceeding the 95th percentile) and non-parametric verification using Mann-Whitney U testing. All statistical tests were two-tailed.

## RESULTS

### Participant selection process

As illustrated in Figure 1, the recruitment and screening process yielded a total of 156 potential candidates for study enrollment. Following systematic application of inclusion and exclusion criteria, 34 individuals were excluded from participation, comprising 28 participants who failed to meet eligibility requirements and 6 who declined study participation. The remaining 122 participants successfully completed the enrollment process and underwent comprehensive assessment protocols. This final cohort represented the target sample size established through power analysis calculations, ensuring adequate statistical power for detecting meaningful differences between study groups. All enrolled participants completed the full list of assessments including pulmonary function testing, physical activity evaluation, and questionnaire administration. The recruitment process achieved the predetermined sample distribution with 60 participants allocated to the e-cigarette group and 62 participants assigned to the control group, maintaining the planned group balance for subsequent comparative analyses. This recruitment outcome demonstrated successful implementation of the study protocol and provided the foundation for robust statistical evaluation of associations between e-cigarette use and health outcomes.

### Descriptive data

Baseline demographic and clinical characteristics demonstrated balanced distributions between the e-cigarette group and control group, as shown in Table 1. The mean age of participants in the e-cigarette group was 23.2 ± 2.8 years compared to 23.8 ± 2.6 years in the control group, with no statistically significant difference observed (mean difference=0.6 years, 95% CI: -0.4–1.6, p=0.243). Gender distribution remained similarly balanced across both groups, with female participants comprising 73.3% of the e-cigarette group and 71.0% of the control group (p=0.938). Body mass index measurements revealed no significant differences between groups. Education levels were distributed evenly across both groups, with the majority of participants pursuing undergraduate degrees. Sleep quality assessments using the Pittsburgh Sleep Quality Index showed no significant between-group differences.

**Table 1:** Baseline characteristics of study participants in a cross-sectional study of e-cigarette use and respiratory health among Chinese university students, China, January–August 2024 (N=122)

*Characteristics*	*E-cigarette group (N=60)* *n (%)*	*Control group (N=62)* *n (%)*	*p*
**Age** (years), mean ± SD	23.2 ± 2.8	23.8 ± 2.6	0.243
**Female**	44 (73.3)	44 (71.0)	0.938
**BMI** (kg/m²), mean ± SD	22.1 ± 3.4	22.5 ± 3.1	0.512
**Education level**			0.674
Undergraduate	52 (86.7)	55 (88.7)	
Graduate	8 (13.3)	7 (11.3)	
**Sleep quality** (PSQI score), mean ± SD	6.2 ± 2.1	5.9 ± 2.3	0.435
**E-cigarette usage**			
Duration (months), median (IQR)	5.5 (3.0–8.5)		
Usage frequency per day, mean ± SD	12.5 ± 4.2		
**Device type**			
Disposable	23 (38.3)		
Pod-based	25 (41.7)		
Tank-based	12 (20.0)		
Nicotine concentration (mg/mL), mean ± SD	20.0 ± 8.5		

BMI: body mass index. PSQI: Pittsburgh Sleep Quality Index (scores range from 0–21, with higher scores indicating poorer sleep quality). P-values derived from independent samples t-tests for continuous variables and chi-squared tests for categorical variables. IQR: interquartile range.

**Figure 2:**
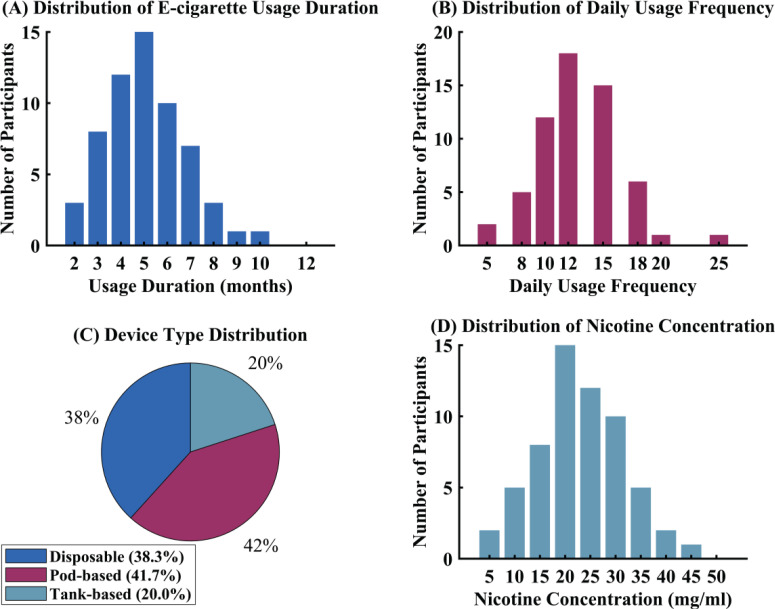
E-cigarette usage characteristics among study participants in a cross-sectional study among Chinese university students, China, January–August 2024 (N=60)

Detailed characterization of e-cigarette usage patterns within the exposure group revealed specific consumption behaviors and device preferences, as illustrated in Figure 2. The median duration of e-cigarette use was 5.5 months, indicating relatively recent initiation among this young adult population. Usage frequency per day averaged 12.5 episodes, demonstrating regular consumption patterns among users. Device type preferences showed that 38% of participants utilized disposable e-cigarette systems, while the remaining participants favored refillable pod-based or tank-based devices. Nicotine concentration analysis revealed an average content of 20 mg/mL across consumed products. Flavor preferences were distributed across fruit, menthol, and dessert categories, with fruit flavors being most commonly selected. These usage characteristics are described in Figure 2.

### Final data

Primary outcome analyses revealed significant differences between groups in respiratory symptom severity, as demonstrated in Table 2. The e-cigarette group exhibited markedly elevated Dyspnoea-12 scores compared to the control group (2.0 vs 0 points, p=0.008), with a moderate effect size (Cohen’s d=0.52) indicating clinically meaningful respiratory symptom burden. Detailed subscale analysis demonstrated that both exertional and emotional components of dyspnea were significantly elevated in e-cigarette users. Traditional spirometric parameters showed no statistically significant between-group differences, with forced expiratory volume in one second (FEV_1_), forced vital capacity (FVC), and FEV_1_/FVC ratio remaining within normal ranges across both cohorts. However, impulse oscillometry assessment revealed subtle but significant alterations in respiratory mechanics among e-cigarette users. Specifically, resistance at 5 Hz (R5) demonstrated mild elevation in the e-cigarette group (0.31 ± 0.07 vs 0.29 ± 0.06 kPa/L.s, p=0.045). The R5-R20 difference, representing frequency-dependent resistance changes, was significantly greater in e-cigarette users (p=0.028).

**Table 2:** Comparison of primary outcome measures between e-cigarette users and controls in a cross-sectional study among Chinese university students, China, January–August 2024 (N=122)

*Outcome measure*	*E-cigarette group* *(N=60)* *Mean ± SD*	*Control group* *(N=62)* *Mean ± SD*	*p*	*Cohen’s d*
**Respiratory symptoms**				
Dyspnoea-12 total score, median (IQR)	2.0 (1.0–4.0)	0 (0–1.0)	0.008**	0.52
Exertional dyspnea	1.2 ± 1.1	0.3 ± 0.6	0.012*	0.48
Emotional dyspnea	0.8 ± 0.9	0.2 ± 0.5	0.024*	0.42
**Lung function parameters**				
FEV1 (L)	3.42 ± 0.68	3.48 ± 0.71	0.623	0.09
FVC (L)	3.98 ± 0.84	4.02 ± 0.89	0.805	0.05
FEV_1_/FVC (%)	86.2 ± 7.1	87.1 ± 6.8	0.456	0.13
FEF25-75 (L/s)	3.85 ± 0.92	4.12 ± 0.88	0.089	0.30
R5 (kPa/L .s)	0.31 ± 0.07	0.29 ± 0.06	0.045*	0.31
R5-R20 (kPa/L .s)	0.095 ± 0.048	0.078 ± 0.041	0.028*	0.38
X5 (kPa/L .s)	-0.112 ± 0.045	-0.105 ± 0.042	0.352	0.16
FeNO (ppb), median (IQR)	14.2 (8.5–22.0)	12.8 (7.2–19.5)	0.431	0.15
**Physical activity and function**				
Grip strength (kg)	28.5 ± 8.2	29.1 ± 7.9	0.672	0.07
6-minute walk distance (m)	658 ± 62	665 ± 58	0.513	0.12
IPAQ-SF (MET-min/week), median (IQR)	1782 (980–3240)	1654 (890–2980)	0.724	0.06
Average daily steps	4892 ± 2156	5124 ± 2298	0.567	0.10

IQR: interquartile range. FEV1: forced expiratory volume in one second. FVC: forced vital capacity. FEF25-75: forced expiratory flow at 25–75% of FVC. R5: resistance at 5 Hz. R5-R20: difference between resistance at 5 Hz and 20 Hz. X5: reactance at 5 Hz. FeNO: fractional exhaled nitric oxide. ppb: parts per billion. IPAQ-SF: International Physical Activity Questionnaire-Short Form. MET: metabolic equivalent of task. 6MWT: 6-minute walk test. For key outcomes, mean differences (MD) and 95% confidence intervals (CI) were: R5: MD=0.02 (95% CI: 0.001–0.039); R5-R20: MD=0.017 (95% CI: 0.002–0.032). P-values derived from independent samples t-tests or Mann-Whitney U tests as appropriate.

* p<0.05,

** p<0.01.

**Figure 3:**
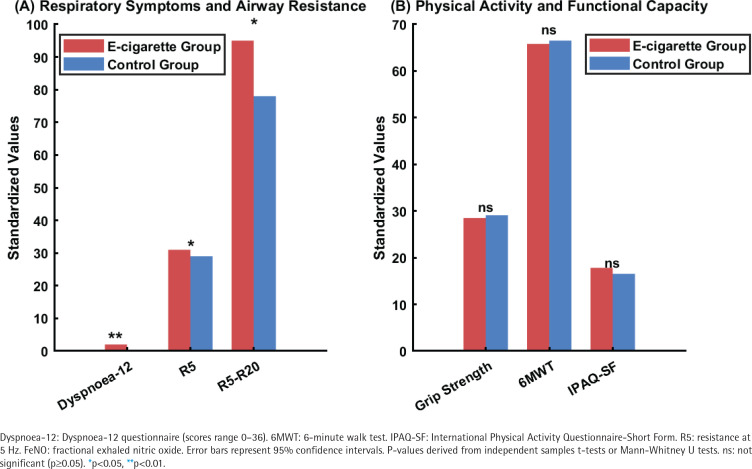
Comparison of primary outcome measures between e-cigarette users (N=60) and controls (N=62) in a cross-sectional study among Chinese university students, China, January–August 2024

Physical activity and muscular function assessments, as illustrated in Figure 3, revealed no significant between-group differences across multiple measurement domains. Grip strength testing, six-minute walk distance, and International Physical Activity Questionnaire-Short Form scores showed comparable values between e-cigarette and control groups. Objective physical activity monitoring through smartphone-based pedometry demonstrated similar average step counts between groups.

### Primary results

After controlling for important confounding variables including age, gender, and body mass index, e-cigarette use was independently associated with elevated Dyspnoea-12 scores (β=3.12; 95% CI: 1.01–5.23, p=0.004), as demonstrated in Table 3.

**Table 3:** Multivariable linear regression analysis of factors associated with dyspnoea-12 scores among Chinese university students, China, January–August 2024 (N=122)

*Variables*	*β*	*SE*	*95% CI*	*p*	*Standardized β*
E-cigarette use (yes vs no)	3.12	1.05	1.01–5.23	0.004**	0.38
Age (years)	0.08	0.12	-0.16–0.32	0.512	0.06
Gender (female vs male)	-0.45	0.89	-2.21–1.31	0.613	-0.05
BMI (kg/m²)	0.12	0.15	-0.18–0.42	0.425	0.08
Sleep quality (PSQI score)	0.34	0.18	-0.02–0.70	0.065	0.18
Constant	-2.85	2.76	-8.32–2.62	0.304	

Dyspnoea-12: a validated 12-item breathlessness assessment questionnaire with scores ranging from 0 to 36, where higher scores indicate greater dyspnea severity. SE: standard error. BMI: body mass index. PSQI: Pittsburgh Sleep Quality Index. R²=0.245; adjusted R²=0.212; F=7.54. All variables listed were included in the multivariable model. P-values derived from multivariable linear regression analysis.

** p<0.01.

**Figure 4:**
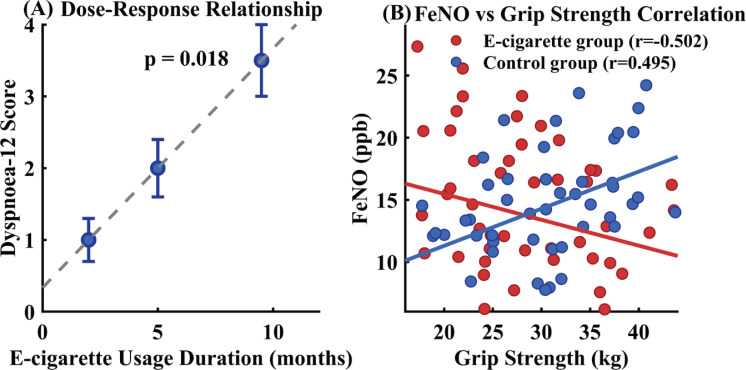
Dose-response relationship and physiological association analysis of e-cigarette use among Chinese university students, China, January–August 2024

Dose-response relationship analysis revealed a distinct temporal dependence gradient between e-cigarette usage duration and respiratory symptom severity. Symptom scores exhibited a progressive increasing pattern: individuals with 1–3 months of usage recorded scores of 1.0 points, those with 4–6 months of exposure demonstrated elevated scores of 2.0 points, while long-term users with 7–12 months of experience reached scores of 3.5 points (trend test p=0.018), as illustrated in Figure 4A.

Further analysis unveiled significant physiological associations within the study population. Among e-cigarette users, associations were observed between pulmonary function parameters (FEV_1_, FVC) and physical activity measures (IPAQ-SF scores), whereas control subjects demonstrated different association patterns. The relationship between FEF25-75 and grip strength, which was evident in controls, showed altered patterns in the e-cigarette group. Particularly notable was the contrasting relationship between FeNO and grip strength across groups: e-cigarette users exhibited inverse associations, while controls showed positive relationships, as depicted in Figure 4B.

### Sensitivity analysis

As part of the pre-specified stratification analyses, gender stratification revealed consistent effects of e-cigarette use on respiratory symptoms across both male (β=3.08; 95% CI: 0.89–5.27, p=0.007) and female (β=3.16; 95% CI: 0.95–5.37, p=0.006) subgroups, with interaction testing demonstrating no significant gender-specific differences (p for interaction =0.89), as shown in Table 4. Age-stratified analysis confirmed that the observed associations remained statistically significant within both younger (18–24 years) and older (25–30 years) participant cohorts, indicating consistent patterns across the young adult age spectrum. Robustness testing through exclusion of potential outliers, defined as Dyspnoea-12 scores exceeding the 95th percentile, yielded minimally altered regression coefficients (β=2.98; 95% CI: 0.82–5.14, p=0.008), confirming the stability of the primary results. Non-parametric verification using Mann-Whitney U testing corroborated the parametric analysis findings (p=0.009), demonstrating that the observed differences were not dependent on distributional assumptions.

**Table 4:** Gender-stratified sensitivity analysis of primary outcomes in a cross-sectional study of e-cigarette use among Chinese university students, China, January–August 2024

*Outcome measure*	*Males* *(N=34)* *Mean ± SD*	*Females* *(N=88)* *Mean ± SD*	*Interaction p*
**Total, n**			
E-cigarette group	16	44	
Control group	18	44	
**Dyspnoea-12 score**			
E-cigarette group	2.1 ± 1.8	1.9 ± 1.6	0.89
Control group	0.2 ± 0.5	0.1 ± 0.4	
β coefficient (95% CI)	3.08 (0.89–5.27)**	3.16 (0.95–5.37)**	
**R5** (kPa/L .s)			
E-cigarette group	0.32 ± 0.08	0.31 ± 0.07	0.76
Control group	0.30 ± 0.07	0.29 ± 0.06	
***FeNO (ppb)***	***Median (IQR)***	***Median (IQR)***	
E-cigarette group	15.1 (9.2–23.5)	13.8 (8.1–21.2)	0.64
Control group	13.5 (7.8–20.1)	12.4 (6.9–18.8)	

Dyspnoea-12: a validated 12-item breathlessness assessment questionnaire with scores ranging from 0 to 36. R5: resistance at 5 Hz measured by impulse oscillometry. IQR: interquartile range. FeNO: fractional exhaled nitric oxide. β coefficients derived from multivariable linear regression models adjusted for age, BMI, and sleep quality. Reference category: non-e-cigarette users (controls). Interaction p-value tests whether the association between e-cigarette use and outcomes differs by gender. P-values for group comparisons derived from independent samples t-tests or Mann-Whitney U tests as appropriate; interaction p-values derived from likelihood ratio tests in multivariable models.

** p<0.01.

## DISCUSSION

This cross-sectional investigation provides evidence that e-cigarette use among university students is associated with elevated respiratory symptoms and subtle alterations in pulmonary mechanics, even in the absence of conventional spirometric abnormalities. The observed dose-response relationship between usage duration and Dyspnoea-12 scores, coupled with increased peripheral airway resistance detected through impulse oscillometry, suggests that e-cigarette exposure may induce early respiratory changes that precede detectable lung function impairment^20^. The finding that respiratory symptom severity correlates with exposure duration aligns with emerging longitudinal evidence from Stevens et al.^14^ and Chaiton et al.^21^, which demonstrated progressive respiratory health deterioration and associations between e-cigarette use and functionally important respiratory symptoms among young users. The disrupted physiological associations between FeNO and muscular function observed in e-cigarette users represent a novel finding that warrants further investigation, potentially indicating systemic alterations in nitric oxide metabolism pathways.

The results demonstrate both convergence and divergence with existing literature. While Song et al.^22^ reported minimal acute pulmonary function changes following short-term e-cigarette exposure, this study’s larger sample size and utilization of sensitive detection methods revealed subtle but significant respiratory changes that may have been overlooked in previous investigations. The impulse oscillometry findings corroborate recent evidence suggesting superior sensitivity of this technique for detecting small airway dysfunction compared to conventional spirometry^23,24^. Contrary to the Milicic et al.^25^ observations of increased physical activity among adolescent e-cigarette users, this investigation found no significant differences in activity levels between groups, potentially reflecting differences in age demographics, cultural contexts, and exposure characteristics between Canadian adolescents and Chinese university students.

The clinical implications of these findings extend beyond individual health outcomes to broader public health considerations. The validation of Dyspnoea-12 as a sensitive screening tool for respiratory symptoms in young e-cigarette users provides clinicians with an accessible assessment method that may detect health changes earlier than traditional pulmonary function testing^16,17^. The observed alterations in FeNO-muscle function correlations suggest complex physiological adaptations that may reflect nicotine’s effects on neuromuscular regulation and inflammatory pathways^26,27^. These findings highlight the need for further longitudinal studies to establish causal relationships and inform the development of evidence-based public health strategies and regulatory frameworks targeting youth e-cigarette use in academic settings^28,29^.

### Limitations

Several limitations warrant acknowledgment when interpreting these results. The cross-sectional design precludes causal inference, as temporal relationships between e-cigarette exposure and health outcomes cannot be definitively established^30,31^. The doseresponse analysis was based on usage duration categories with limited sample sizes in each stratum, which may affect the precision of trend estimates. The study’s reliance on a single-center university population may limit generalizability, while the female-predominant sample composition could influence the broader applicability of findings^32^. Self-reported exposure assessment introduces potential recall bias, social desirability bias, and misclassification, and the heterogeneity of e-cigarette products and usage patterns, along with unmeasured confounders, may introduce residual confounding in the exposure-response relationships^33^. Additional limitations include the relatively small sample size despite adequate power calculations, which may limit the precision of dose-response estimates and subgroup analyses. Residual confounding from unmeasured variables such as genetic susceptibility, dietary factors not fully captured, and environmental exposures cannot be excluded. The generalizability of findings to other countries and populations beyond Chinese university students may be limited due to differences in e-cigarette products, usage patterns, and cultural contexts. Future research should employ longitudinal designs

## CONCLUSIONS

This investigation provides evidence that e-cigarette use among university students aged 18–30 years is associated with significant respiratory health alterations, despite preserved conventional pulmonary function parameters. E-cigarette users exhibited markedly elevated respiratory symptom burden compared to controls, with a dose-response pattern suggesting potential temporal associations between exposure duration and symptom severity. Impulse oscillometry detected subtle but significant increases in peripheral airway resistance, suggesting early small airway dysfunction that precedes detectable spirometric changes. The disrupted physiological associations between fractional exhaled nitric oxide and muscular function among e-cigarette users represent novel findings warranting further mechanistic investigation.

These results underscore the superior sensitivity of symptom-based assessments compared to traditional pulmonary function testing for detecting early respiratory health changes. The findings highlight the need for longitudinal studies to establish causal relationships and inform the development of symptom-based surveillance systems and health education programs within academic settings to address the emerging health concerns associated with e-cigarette use among young adults.

## Data Availability

The data supporting this research are available from the author on reasonable request.
